# Empirical Insights of Individual Website Adjustments for People with Dyslexia

**DOI:** 10.3390/s19102235

**Published:** 2019-05-14

**Authors:** Katja Kous, Gregor Polančič

**Affiliations:** Faculty of Electrical Engineering and Computer Science, University of Maribor, Koroška cesta 46, 2000 Maribor, Slovenia; gregor.polancic@um.si

**Keywords:** web accessibility, usability testing, assistive technology, individual website adjustments, customised websites, people with dyslexia

## Abstract

The existing body of knowledge reveals that customisable websites may lead to an increase in accessibility and usability for people with disabilities. In this way, the main goal of this research was to investigate how people with dyslexia respond to a customised version of a website in terms of its effectiveness, efficiency, satisfaction and suitability when compared to the default version of the website. The customisation of the investigated website was enabled with the aid of integrated assistive technology that offers people with dyslexia the opportunity to adjust a website themselves in accordance with their individual needs, demands and preferences. They can do this by changing the parameters, such as font size, font type and contrast between the background and text. The answers to the research questions were obtained with complementary research methods and techniques, including formal usability testing, thinking aloud protocol, log analyses, questionnaires and interviews. The empirical results show that participants experienced more issues when interacting with the default website, and they enjoyed more benefits when using the customised website. Too much information on the screen, not enough graphic elements, issues with visual appearance and inappropriately presented information were identified as the most common issues when interacting with the default website. When using the customised website, all participants agreed on a better user experience and, as the majority of them reported, this was due to appropriate contrast and font size. Additionally, the majority of participants also expressed desire to use the individual website adjustments regularly in the future. The conclusions of this investigation are that the individual website adjustments used in this research can not only help to minimise issues, but also eliminate challenges that people with dyslexia have when interacting with a website. Therefore, the primary contributions of this research are the empirical insights of interaction with both the default and customised version of the website for people with dyslexia. Furthermore, this research also has three secondary contributions: (1) detailed presentation and application the general usability evaluation procedure to a specific target group (people with dyslexia); (2) recommendations to adapt the usability evaluation methods for people with dyslexia; and (3) the usage of quantitative measurement instruments for the evaluation of a website’s usability and suitability for people with dyslexia.

## 1. Introduction

Information technology has an important role in the lives of people with disabilities [1], because it enables them to better integrate into society [2]. However, to ensure integration, it is necessary to increase efforts to adapt information and communication technologies so that they can be used by everyone, including those who have some form of disability [2]. To ensure equal access and equal opportunities for using websites, tools, and technologies for all people, the term “web accessibility” was formulated [3]. This states that all people (including people with visual, auditory, physical, speech, cognitive, and neurological disabilities) can perceive, understand, navigate, interact and contribute to the Web [3]. To ensure web accessibility, many guidelines and standards have been proposed, such as ISO/IEC 40500:2012 [4], Web Content Accessibility Guidelines (WCAG 2.1) [5], Section 508 [6], Design for all [7], etc. The common denominator of these recommendations is that they provide instructions to designers and developers about how to create and develop a website that is useful for a multitude of people with different abilities, needs, demands and preferences (hereinafter “needs and preferences”).

Users’ needs and preferences can be diverse not only in terms of different end user groups (e.g., blind people and deaf people), but also within end-user groups (i.e., within the group of people with cognitive disabilities). It means that what is appropriate for one group or for one representative of a group may not be appropriate for another group or for another representative from the same group. This research focused on people with different needs and preferences within the group, more specifically on people with dyslexia. The peculiarity of this group is that it is not possible to define a universal profile of people with dyslexia [8,9,10], because each person within the group has different experiences with the manifestations of dyslexia.

Dyslexia is one of the most common learning disabilities [11] that primarily affects reading, spelling and writing skills as well other communication-related cultural abilities. According to the International Dyslexia Association [12], 15–20% of the global population has at least one of the symptoms of dyslexia, such as slow or inaccurate reading, difficulty spelling, low proficiency in writing, a tendency to mix up similar letters or words. In Europe, the percentage of people with dyslexia is lower and encompasses between 5% and 12% of the population [13]. Although signs of dyslexia vary between people and difficulties can manifest themselves in different areas and with varied intensity, some common characteristics among people with dyslexia are defined, and include difficulties with phonological processing (the manipulation of sounds), spelling, and/or rapid visual-verbal responding [11]. The complete set of possible problems that generally characterise people with dyslexia can be divided into six areas according to Woodfine et al. [14]: reading, writing, speaking, memory, organisational and general. Because of their individuality, there is no universal profile for people with dyslexia [8,9,10]. Furthermore, there is also no universal dyslexic web user [8]. Therefore, recommendations for designing universal websites can be challenging because a single design may not work for everyone due to the different needs and preferences of individuals with dyslexia [15]. Therefore, some authors recommend using a customisable web environment [9,16] with the possibility of website transformation [17,18] in accordance with users’ individual needs and preferences.

Personalisation and customisation are well-known strategies and they have also been implemented in order to improve access to the Web and to improve the user experience [17,19]. The existing literature reports some solutions for the personalisation and customisation of websites for people with dyslexia, such as web-based services [20], a plugin for browsers [21] and transcode techniques [17]. This research focused on the customisation of website in accordance with users’ individual needs and preferences. The customisation of website was enabled via integrated assistive technology, which offers users the opportunity of adjusting websites by themselves by changing the parameters (such as font size, font type and contrast between the background and text). The values collections of individual website adjustments were selected in accordance with recommendations for people with dyslexia. Therefore, the individual website adjustments provides a customised website that is in accordance with user’s individual needs and preferences.

Some studies reports that a customised website may lead to increases in accessibility as well as usability [18,22,23], including for people with dyslexia [9,24]. Based on that assumption, this research focused on empirical advantages of customised websites when compared to a default website. More specifically, the research investigated the usability and suitability of customised websites for people with dyslexia when compared to a default website. The construct of usability was treated as “the extent to which a product can be used by a specified user to achieve specified goals with effectiveness, efficiency and satisfaction in a specified context of use” [25]. The construct suitability was defined as “the degree of appropriateness of the system to the tasks which have to be accomplished” [26] (in this research, the system means website), taking into account the user’s individual needs and preferences. In addition, the research also verified if the people with dyslexia selected different individual adjustments. The answers to the research questions were obtained by performing formal usability testing, the thinking aloud protocol, log analyses, questionnaires and interviews.

## 2. Literature Review and Research Questions

The symptoms of dyslexia vary from person to person. According to existing literature, there are a set of parameters for text presentation (e.g., font size and type, character spacing, line spacing, paragraph spacing, and colours), which may lead to increases in readability for people with dyslexia. Furthermore, Rello et al. [27] found that texts presented with several synonyms for a complex word are more readable and comprehensible for people with dyslexia. Additionally, there is also no universal dyslexic web user [8]. The one way to increase web accessibility for different profiles of people with dyslexia is to offer them the opportunity of transforming a website in accordance with their individual needs and preferences. Santana et al. [16] reported that people with dyslexia have different abilities and different preferences regarding color, font type and size. They recommended offering people with dyslexia the opportunity to customise the website through a selection of font style and size, background and font colour [16]. Evett and Brown [28] also stated that the opportunity to select the font style, font size, background and text colour is one of the recommendations for website design for people with dyslexia. Based on these findings, this research focused on the following parameters: font size, font type and contrast between the background and text.

**Figure 1:**
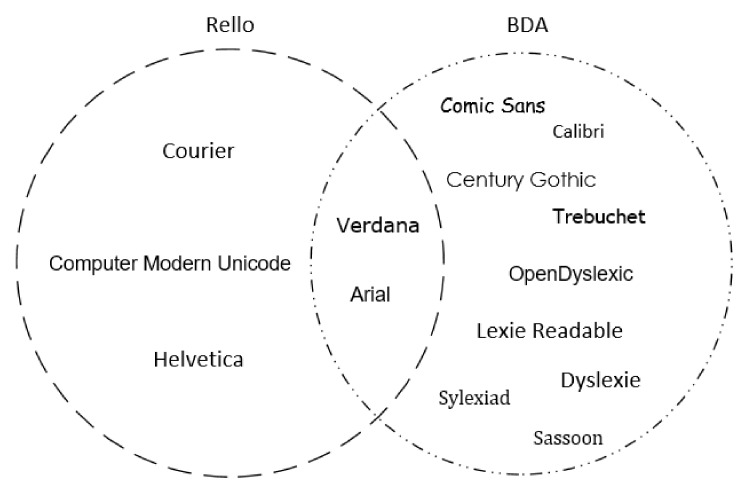
Font types recommended by Rello [20,32] and BDA [29].

Many recommendations are published by different associations for people with dyslexia [12,29,30] and many researchers [20,24,27,28,31,32,33,34,35,36] recommend guidelines for a dyslexic friendly website including the preferred choices in accordance with their needs and preferences. The British Dyslexia Association [29], Bradford [31] and Zarach [35] agree that the most adequate font size is 12 or 14 points. Rello [20] pointed out that a bigger font size leads to improved text readability. citetRello2016a found that a local maximum for objective readability is between 18 and 26 points. In the literature, the Arial font is highly recommended by different associations [29,30] and researchers [20,28] because it has the shortest reading time for people with, and also without, dyslexia. The multitude of font types recommended by the relevant literature is displayed in Figure 1. The British Dyslexia Association [29] recommends using the type font group “sans-serif”, while Rello and Baeza-Yates [32] additionally recommend “roman” and “monospaced”. Rello and Bigham [34] found that background colours have an impact on the readability of text for people with dyslexia. Therefore, the warm background colours (Peach, Orange and Yellow) significantly improved reading performance over cool background colours (Blue, Blue Grey and Green) [34]. According to Bradford [31], many people with dyslexia are sensitive to high colour contrasts, therefore the author does not recommend black text on a white background. Rello [20] stated that people with dyslexia prefer a high contrast between the text colour and background, such as the combinations of a mucky green text on a brown background and blue text on a yellow background. Gregor and Newell [24] suggested that using brown text on a mucky green background and blue text on a yellow background, while Evett and Brown [28] recommend the use of a black text on a yellow background or dark-blue text on a pale-blue background.

To summarise, there is no universal profile of characteristics of people with dyslexia and they have different abilities and different preferences regarding parameters of individual adjustments, such as colours, type and sizes [16]. Based on this assumption, the research question was defined: 

**RQ1:** What parameters of individual website adjustments for people with dyslexia diverge or converge? 

The existing literature focuses on identifying and eliminating the problems that people with dyslexia encounter when using a website. While some of the research investigated how to present more readable text for people with dyslexia [9,37,38,39], others explored the main challenges with web interaction within a group of people with dyslexia [37,40,41,42,43,44,45,46,47]. The inappropriate text presentations have negative effects on reading speed, comprehension, satisfaction as well as on the level of reading difficulty [37]. In addition, people with dyslexia usually encounter website navigation problems. Pieper [46] found that they have orientation problems when navigating within a website. Al-Wabil et al. [41] investigated experiences with web navigation for people with dyslexia. They revealed several patterns of behaviour, including frustrations with website structures and textual presentations. Participants’ navigation trails varied based on the severity of their reading impairment. The less impaired users stated that navigation trails helped them to understand their location, while moderately impaired users stated that navigation trails were helpful for backtracking, but not for keeping track of their location within a site [41]. Freire et al. [42] found that people with dyslexia did not share a single preferred strategy. They usually use a strategy where they relate to moving from one page to another, followed by the strategy of returning to home pages and the strategy of using keyword searches to find the content in a website. The next challenge for people with dyslexia when using a website is inappropriate elements layout, that, together with too small fonts and inappropriate colour background, could lead to reduced web accessibility [43]. According to Smythe [40], issues can be divided into four main categories: technological, navigational, typographic and layout ones. Hollins and Foley [45] divided the reasons for the failure of website interaction into six categories: webpage appearance, website structure, navigation, input elements, language and availability of help features. Freire et al. [42] report that the most frequent problems were related to navigation issues, problems with presentation and organisation of information, lack or malfunctioning of a specific functionality within websites, and issues with language. Research [44] shows that following problems defined by people with dyslexia are common to most internet users: confusing page layout, unclear navigation, poor colour selections, graphics and text too small as well as complicated language.

Therefore, people with dyslexia often encounter problems when using a website that is not designed in accordance with their needs and preferences. Based on this assumption, the research investigated if the above-mentioned problems could be eliminated by using a customised website. The corresponding research question was defined as the following: 

**RQ2:** What are the benefits or challenges for people with dyslexia when interacting with adjusting a customised website? 

Some studies reported that a customised website could lead to an increase in usability for people with dyslexia [9,24], but existing research has not fully investigated usability and suitability simultaneously with regard to default and customised websites. By considering this, we focused on the advantages of usability and suitability of a customised website when compared to the default version of a website.

Usability was treated as “the extent to which a product can be used by a specified user to achieve specified goals with effectiveness, efficiency and satisfaction in a specified context of use” [25], where ISO 9241 defines the underlying usability concepts as follows [25]:

- **Effectiveness** as “accuracy and completeness with which specified users can achieve specified goals in particular environments“.
- **Efficiency** as “resources spent by a user in order to ensure accurate and complete achievement of the goals“.
- **Satisfaction** as “the comfort and acceptability of the work system to its users and other people affected by its use“.

Suitability is defined as “the degree of appropriateness of the system to the tasks which have to be accomplished” [26] (in this research, the system means website), taking into account the user’s individual needs and preferences. It consisted of six concepts, identified based on a synthesis of knowledge after a review of existing literature that included: (1) the identification of common characteristics of people with dyslexia; (2) issues that people with dyslexia have when using the website; (3) recommendations for improving web accessibility for people with dyslexia; and (4) recommendations for web design for people with dyslexia. These concepts are:

- **Attractiveness and colour suitability**: Perceived visual attractiveness is defined as “the degree to which a person believes that the website is aesthetically pleasing to the eye” [48]. The visual attractiveness of the website refers to its visual elements, most notably the colours used and its overall layout [48]. Inappropriate colour contrasts between text and background can cause issues with web content for people with dyslexia. The combination of attractiveness and colour suitability means that people with dyslexia perceive and evaluate the website as attractive, despite the individual selection of colour contrast.
- **Content presentation**: The web content refers to any part of a website, including text, images, forms, and multimedia [49]. For people with dyslexia, it is important how the text is designed, because the presentation of the text has a significant effect on the speed of reading for people with dyslexia [9]. An appropriate font size, font type, character spacing, paragraph spacing, line spacing and column width [9,20,37,38,39,40] can lead to improvements in terms of the readability of the text and consequently understandability of the content. It is also beneficial to use images, charts and pictures to complement textual information [16].
- **Navigation and layout**: The web page layout and navigation structure are important website features for people with dyslexia. The existing literature states that an inappropriate web page layout [40] as well as unclear navigation [21,41,42,43,46] can lead to issues for people with dyslexia. Thus, the web page layout should be simple and clean, whereas the navigation should be easy and consistent, visible all the time, and should contain simple lists of link [29].
- **Individual suitability**: The individual suitability relates to the suitability of a website that is consistent with users’ expectations, needs and preferences. Because no universal dyslexic web user can be specified [8], it is even more important that the website is designed in accordance with the individual needs of people with dyslexia.
- **Ease of use**: Davis [50] defined “ease of use” as “the degree to which a user believes that using a website would be easy and free of effort”. Because of some of the symptoms that people with dyslexia have (such as problems with working memory and/or orientation) they need a website that is easy for them to use [29].
- **Learnability**: Nielsen [51], as many other experts [52,53,54,55,56], defined learnability as one of the concepts of usability. It asks: “How easy is it for users to accomplish basic tasks the first time they encounter the design?” [51]. People with dyslexia can have problems with short-term memory, or, more specifically, working memory, thus it is important that the website is not only easy to use but also easy to learn.

Therefore, the third research question was defined as follows: 

**RQ3:** Do individual website adjustments for people with dyslexia yield any empirical advantages related to effectiveness, efficiency, satisfaction and suitability? 

The answers to the research questions represented in this section were obtained by performing formal usability testing, thinking aloud protocol, log analyses, questionnaires and interviews. The research details are represented in the following section.

## 3. Empirical Research

### 3.1. Research Goal

The main goal of this research was to investigate how people with dyslexia respond to a customised version of a website in terms of its usability and suitability when compared to the default version of a website. The usability was specified as “the extent to which a product can be used by a specified user to achieve specified goals with effectiveness, efficiency and satisfaction in a specified context of use” [25], while the suitability was defined as “the degree of appropriateness of the system to the tasks which have to be accomplished” [26] (in this research, the system means website), taking into account the user’s individual needs and preferences.

### 3.2. The Investigated Website

**Figure 2:**
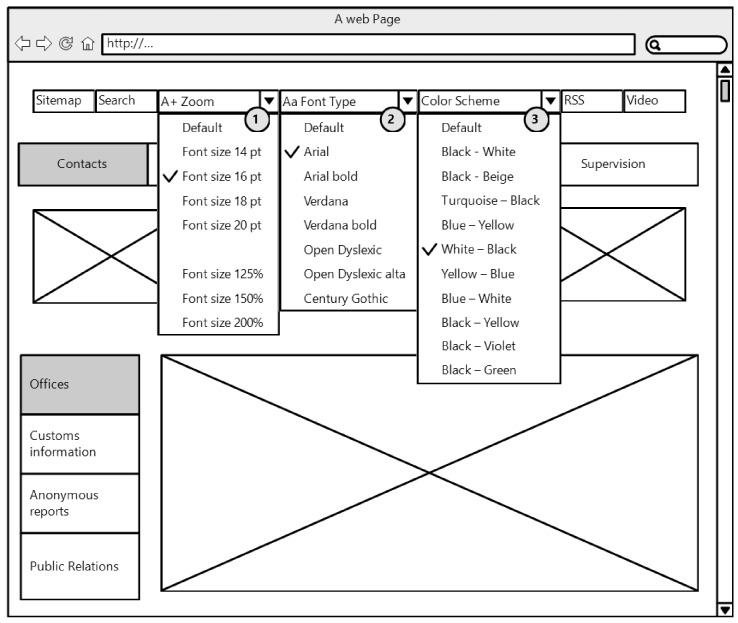
The wireframe of the website with the integrated assistive technology.

The object of the research was a website with integrated assistive technology which offers users the opportunity of adjusting the website by themselves in accordance with their individual needs and preferences by changing the website’s parameters, such as font size, font type and contrast between the background and text. The wireframe of the investigated website is presented in Figure 2.

The first website adjustment “Font size” (No. 1 in Figure 2) offered seven sizes (14 pt, 16 pt, 18 pt, 20 pt, 125%, 150%, and 200%). The values collection of second website adjustment “Font type” (No. 2 in Figure 2) included seven font types, such as Arial, Arial Bold, Verdana, Verdana Bold, Open Dyslexic, Open dyslexic alta and Century Gothic. The last website adjustment “Colour scheme” (No. 3 in Figure 2) offered ten contrasts of the text and background (such as Black-White, Black-Beige, Turquoise-Black, Blue-Yellow, White-Black, Yellow-Blue, Blue-White, Black-Yellow, Black-Violet and Black-Green).

### 3.3. Participants

To invite the target group of participants, which was composed of people with a medically confirmed diagnosis of dyslexia, the direct recruitment and chain sampling were used as recruiting methods. The six participants responded to an invitation to participate in the entire process of the study. In respect to legal and ethical matters, all participants were adults (over 18 years old). While no ethics committee is available in the home institution, the informed consent was obtained from all of them prior to starting with the process of the study. Thus, the research sample consisted of six people with a medically confirmed diagnosis of dyslexia, with no age restrictions. The sample size is in accordance with Nielsen’s findings for sample sizes [57,58,59] and within the recommendations for research with involved users with disabilities [60]. Lazar et al. [60] emphasised that “it is generally acceptable to have 5–10 users with a specific impairment take part in a study”. The demographic characteristics of the participants are shown in Table 1.

The demographic information in Table 1 reveals that three participants (50%) were female and three participants (50%) were male. Three participants (50%) were less than 25 years old, two participants (33%) were between 26 and 35 years old and one (17%) was more than 36 years old. Four participants (67%) had a poor knowledge of assistive technology and two (33%) occasionally used assistive technology, such as enlarging font size and changing the background colour, while the others never used it.

### 3.4. Methods And Metrics

The experiment was based on the following methods: (1) formal usability testing; (2) thinking aloud protocol; (3) log analysis; (4) questionnaires; and (5) interview.

The construct usability was composed of three concepts [25]:

1. Effectiveness represents the percentage of users who successfully achieve each goal of use of the website [61].
2. Efficiency represents the time taken to achieve the goal [61].
3. Satisfaction is the level of the user’s overall satisfaction when they use the website [61].

The construct of suitability was specified out of six concepts:

1. Attractiveness and colour suitability represent the degree of the user’s opinion on the attractiveness and suitability of a website, despite the individual selection of colour contrast.
2. Content presentation is the degree of the user’s opinion, related to a subjective assessment of the appropriateness of the content presentation of information.
3. Navigation and layout is the degree of the user’s opinion related to a subjective assessment of the ease of website navigation experience and the appropriateness of the layout of elements.
4. Individual suitability represents the degree of the user’s subjective opinion on the suitability of the website that is in accordance with their expectations and needs.
5. Ease of use is the degree to which a user believes that using the website would be easy and free of effort [50].
6. Learnability represents the degree of the user’s opinion related to a subjective assessment of simplicity performance of basic tasks when the website is used for the first time [51].

Efficiency was measured by the time task [61,62,63,64], while the effectiveness was measured by successful task completion [61,62] using a three-point scale (where 1 means “Participant completed the task successfully”; 2 means “Participant completed the task successfully with the help of the moderator” and 3 means “Participant provided the wrong answer or gave up before completing the task”). Satisfaction was measured by the standardised questionnaire System Usability Scale (SUS) [61,62] developed by the Digital Equipment Corporation. Participants’ satisfaction was measured by a five-point Likert scale (where 1 indicated that the participants totally disagreed with the sentence, while 5 indicated that the participants totally agreed with it) and then evaluated with the SUS protocol [65]. The suitability questionnaire (Appendix A) was composed of specific questions from different questionnaires related to usability and the accessibility domain. The answers were measured with a five-point Likert scale.

### 3.5. Experimental Environment

In accordance with the recommendations for usability testing procedure [62], the usability test was created and performed by an observational coding system called Morae. All three components of the system were used [62]: (1) Morae Recorder—used for the preparation and execution of usability testing; (2) Morae Observer—used for monitoring events on the participant’s screen and for the management of observational data; and (3) Morae Manager—used to manage data. Participants used the Morae Recorder for the entire time of the usability testing process, including questionnaires. Although the task instructions (Appendix B) were presented in the Morae Recorder and could be displayed during the execution of the task, the task instructions also offered paper-based forms for participants. In accordance with recommendations for people with dyslexia, the paper was white and the font on the paper was blue with 18 point size Arial.

**Figure 3:**
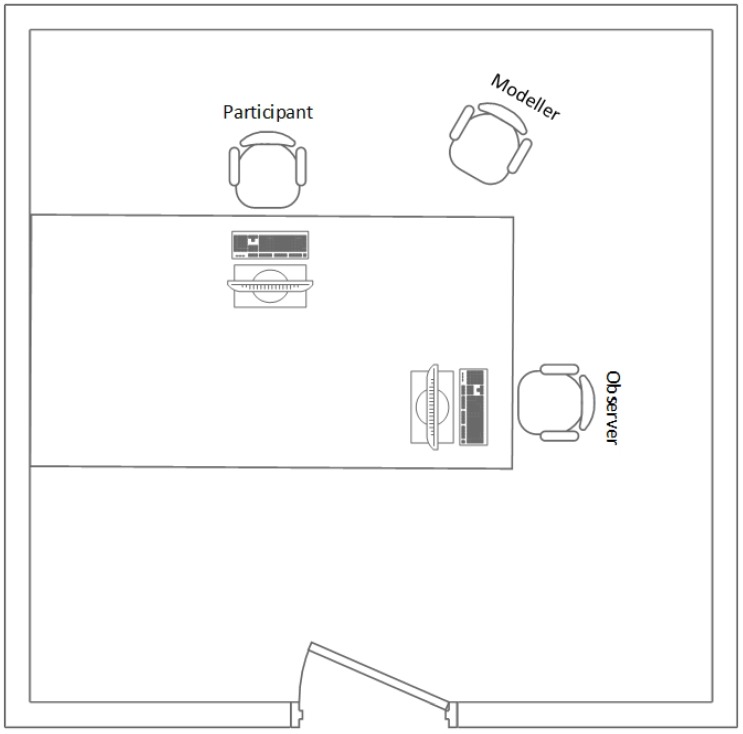
The environment for usability testing.

The experiment was conducted in individual sessions, which were all held in a controlled environment and in the presence of two researchers—the moderator and the observer. The moderator led the whole testing protocol and communicated with participants, while the observer monitored the situation and took notes about all participants’ comments when they were thinking aloud [62]. As illustrated in Figure 3, the environment was adapted to the performance of the usability testing. The testing room was free from distractions. It was comfortable and large enough to accommodate the moderator, observer and participant and the participant had enough privacy for the execution of tasks. In the room were two computers—one for the participant and the other for the observer.

### 3.6. Execution of The Research

Before beginning with the usability evaluation, a pilot test was performed to check for the technical and usability weaknesses of the planned usability testing. The technical check verified if the test was working properly, while the usability check reviewed if the test was easy enough to understand and if it provided the required answers [62]. The technical check showed that the font size for the task instructions was too small for people with dyslexia. Based on this identified weakness, the font size for task instructions was increased from 14 pt to 18 pt. An analysis of the usability check’s data showed that the three task descriptions were not understandable. To improve the understanding of task instructions, the instructions were changed in accordance with recommendations.

After the pilot research, people with a medical confirmed diagnosis of dyslexia were kindly invited to participate in the research. When the participant responded to the invitation, researchers planned an appropriate time for testing. On the scheduled day, the moderator had a welcome speech and presented the instructions for testing to the participant. Each participant was informed that the usability testing was anonymous, and that they were not testing them and their skills, but the usability of the default and customised version of website. To ensure consistency in testing, the participant was asked to reset the browser to “home” after completing each task. If a participant did not have any questions, he/she started with the experiment procedure.

The experiment procedure was performed in three stages. In the first stage, each participant was asked to fill-out the questionnaire with the aim of collecting demographic information. After that, participants started with the second stage, based on formal usability testing with a thinking aloud protocol. This stage was separated into two sub-stages:

1. Sub-stage A involved tasks using a default version of the website and filling-out the questionnaire related to their satisfaction and the suitability of using the original version of the website.
2. Sub-stage B involved tasks using a customised version of the website and filling-out the questionnaire related to their satisfaction and suitability of using the customised version of the website. Before the participants started to perform tasks with the customised version, they had to adjust the default website according to their needs and preferences. At the end of the performed tasks that used the adapted conditions, they had to turn off the adjustments and return the website to its default state.

Each participant was involved in both sub-stages. To cancel out learning and sequence effects, the order of sub-stages was randomised. In the third stage, a two-question interview was conducted. The aim of the first question was to gain the participant’s subjective opinions about both the default and customised version of the website, while the aim of the second one was to obtain information for the future use of the customised version. After performing the entire experiment procedure, each participant received a “thank you” gift.

### 3.7. Data Collection Protocol

The empirical data (i.e., successful task completion, task time, satisfaction level and suitability level) were collected using the formal usability testing and questionnaires when using the Morae Recorder. The qualitative data were collected using the thinking aloud protocol (i.e., participants’ comments), log analysis (i.e., observer’s findings) and interview (i.e., participants’ answers). To facilitate a more detailed analysis of performance tasks, the observer followed and recorded the action on the participants’ screens with a Morae Observer and took notes of the participant’s comments during usability testing. To ensure the anonymity of individual data, shots were saved with an identification number (i.e., No_Participant).

### 3.8. Data Analysis

The data were analysed using quantitative and qualitative methods. The quantitative method was employed to conduct a descriptive data analysis using the statistical tool SPSS (version 24), while the qualitative analysis was executed by reviewing, coding, analysing and interpreting the participants’ comments obtained during usability testing. Data coding and analysing were performed using the software tool for qualitative data, called QDA Miner (version five).

## 4. Results And Discussion

### 4.1. Individual Website Adjustments

The values collections of individual website adjustments were selected in accordance with literature recommendations [20,29]. The participants had to select from three parameters of website adjustment, such as font size, font type and contrast. They received instructions that they had to select the adjustments, which is the most suitable for their needs and preferences. The results are presented in Table 2.

As shown in Table 2, 33% of participants used the font size (the first adjustment’s parameter) 18 pt that significantly improves in readability and comprehensibility according to Rello and Baeza-Yates [9]. The other participants selected the alternative options of smaller font size, such as 14 pt (17%) or 16 pt (17%) or zoom at 125% (17%) or 150% (17%), although Rello and Baeza-Yates [9] pointed out that larger font sizes are more readable for people with dyslexia. Overall 67% of participants selected Arial as the most appropriate font type (the second adjustment’s parameter). In the existing literature, Arial is a highly recommended font for people with dyslexia [9,29,30,38] and has also been described as the font with the shortest reading time [38]. One participant (17%) used Verdana, which is recommended by guidelines [20,29,38]. Surprisingly, only one participant (17%) had already known about, and selected, the specific font for people with dyslexia called Open dyslexic. Zikl et al. [66] even found that the font Open dyslexic did not lead to any marked improvements in reading speed, although a number of participants claimed that Open dyslexic was more readable for them. The last parameter was the adjustment of contrast between font and background. As shown in Table 2, 33% participants selected a black font on a beige background, which is also recommended by British Dyslexia Association [29]. The other preferred colour pairs chosen by participants were: black/white (in accordance with Bradford [31]), blue/yellow (in accordance with Gregor and Newell [24] and British Dyslexia Association [29]), turquoise/black (in accordance with British Dyslexia Association [29]) and white/black (in accordance with Rello [20]).

### 4.2. Benefits and Challenges of Interacting with the Adjusted Website

Table 3 shows the benefits and challenges based on an analysis of the participants’ comments. As detailed in Table 3, most participants reported challenges when interacting with the default version of the website.

The majority of the participants (4/6, 67%) expressed a desire to use the search engine for information re tribal, because the navigation was unclear (e.g., “Very poor navigation. [...] I get lost.”, “I’m lost without a search engine.”, etc.). They also emphasised that this approach is their primary search strategy. This strategy is defined as the third most often used strategy for people with dyslexia [47]. Overall, 67% (4/6) of participants had a problem with information overhead on a single page (e.g., “When there is a lot of text, it’s harder to find the correct information.”, “Too much text on one side. [...] this website is worse than a customized one.”, etc.). Participants also reported insufficient graphic elements (e.g., “The website is less understandable for me, because there are no images.”, “Very disturbing [...] there were no images, I’d rather search by pictures and icons.”, etc.). Although this is in line with existing research, which recommends the use of images and icons rather than words for people with dyslexia [16], Berget et al. [36] found that information presented with icons and words in a list structure does benefit people with dyslexia (and people without dyslexia).

The second most commonly reported problem by different participants was related to unsuitable visual appearances (e.g., “I’m looking for the information. I can not find it [...] the visual presentation is improper for me.”, etc.) and visual complexity (e.g., “I’m a little confused [...] I do not know exactly which one is right.”, etc.), which is also one of the most commonly identified problems in the existing literature [42,45]. Overall, 50% of participants (3/6) reported that these problems in combination with an inappropriate font type, led to the unreadability of text and consequently to a misunderstanding of the content. Overall, 33% of participants (2/6) reported problems with imperfections and the inconsistency of content (e.g., “Unclear description.”, “[...] the structure of the content areas is wrong.”, etc.), inappropriate font types (e.g., “It is hard to read, [...] this is no suitable font for me.”, etc.) and inappropriate contrasts between the background and the font (e.g., “The background is not suitable for me, I prefer a darker background.”, “I’m a little bit confused, [...] this colour is not for me.”, etc.). As reported by Chen and Keong [37], inappropriate text presentations have negative effects on reading speed, comprehension, satisfaction as well as on the level of reading difficulty.

The comments mentioned above were identified as challenges for people with dyslexia when they used a website that was not in accordance with their needs and preferences. The benefit of using the default website was expressed by two participants (33%) who said that the default website included the appropriate font size (e.g., “Font size is good for me.”, “The website is more clear, because the smaller font is used.”, etc.). The same positive experience of using the customised website was reported by four participants (67%) (e.g., “Finally, I can read normally.”, “I need less effort to read, because the letters are bigger.”, etc.). Furthermore, 83% of participants noted an appropriate contrast between the background and font colour (e.g., “It’s easier to find information.”, “The website is more clear, because of the used colour.”, etc.) and all of them reported a better user experience when using the customised website. Nevertheless, participants reported three issues with using the customised website: one participant (17%) expressed the desire to use the search engine, two participants (33%) had a problem with insufficient graphic elements (e.g., “I am missing the graphic elements.”, “I need to read a lot of text, because there are not icons.”, etc.) and one participant (17%) reported problems with visual complexity (e.g., “It was harder to use the customized version than the default website. [...] It is too complex for me.”, etc.).

### 4.3. Empirical Advantages of Individual Website Adjustments

A detailed analysis was made, based on the participants’ comments during the tasks performed with both the default and customised website. The results are presented in Table 4. The participants reported 52 comments for the default and customised website. The most comments were given by Participant 4 (12 comments), followed by Participants 3 and 6 (both nine comments), Participant 2 (eight comments) and Participants 1 and 5 (seven comments).

#### 4.3.1. Effectiveness

The effectiveness was measured by successful task completion. A comparison shows that, on average, participants achieved an 81% success rate with both the default (± 19%) and customised (± 21%) version of the website.

**Figure 4:**
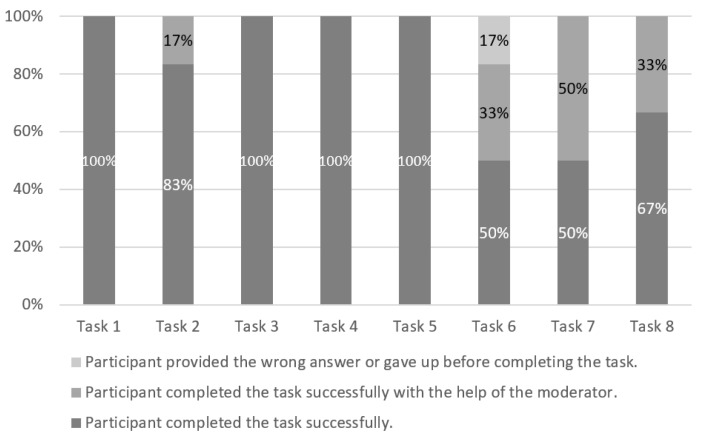
Default version: The levels of success by task.

Figure 4 shows the levels of performed tasks with the used default website, while Table 5 presents the occurrence of participants’ comments during the tasks performed with the default version. The participants had the most issues with Task 6 (50% success rate) and Task 7 (50% success rate) when they used the default version. According to the participants’ comments (Table 5), the potential reason for their low success rate could be information overhead on a single page (Tasks 6 and 7), insufficient graphic elements (Task 6), problems with imperfections and inconsistency of content (Task 6), visual complexity (Task 6) and improper contrast (Task 6). Although the participants reported the majority of comments (nine comments, all identified challenges except information overhead on a single page, visual complexity and improper contrast) during Task 5, they were still able to complete the task by themselves. They also found the correct answers by themselves for Task 1 (six comments), Task 3 (one comment) and Task 4 (one comment).

**Figure 5:**
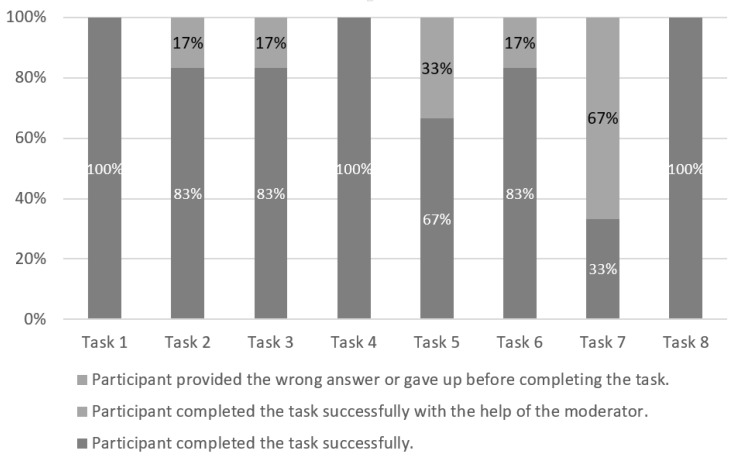
Customised version: the levels of success by task.

Figure 5 presents the levels of performed tasks with the customised website, while Table 6 shows the occurrence of participants’ comments during the task performed with the customised version. When participants used the customised version (Figure 5), they completed three tasks (Tasks 1, 4 and 8) by themselves. While carrying out these tasks they reported that the website included an appropriate font size (Tasks 1 and 8) and appropriate contrast (Tasks 1 and 8). All of them also reported a better user experience (Task 8). Only one comment about insufficient graphic elements was given during the performance of Task 4. Although Task 7 had a success rate under 40%, none of the participants pointed out any of the challenges during a performance of the task.

#### 4.3.2. Efficiency

**Figure 6:**
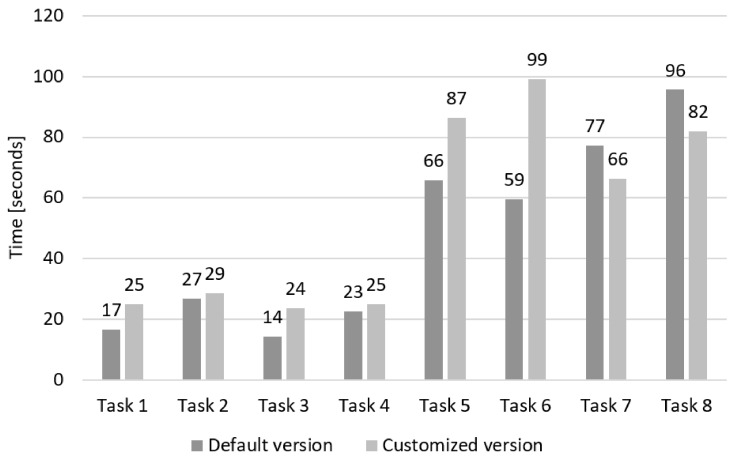
The average time for performing each task, using the default and customised version.

The efficiency was measured by the time needed to finish tasks. The average amount of task time was 47.31 (± 31.17) s per task when using the default version of the website, while the average time for completing one task when using the customised version website was 54.52 (± 32.27) s per task. The average time deviation between the default and the customised version was 13.5 s. As illustrated in Figure 6, the detailed comparison shows that participants needed more time to complete the Tasks 1–6 and less time to complete Tasks 7 and 8 when they used the customised version of the website. The maximum deviations of the task times between the default and the customised website were detected for Task 5 (21 s) and Task 6 (40 s). While performing these tasks, none of the participants pointed out any challenges with using the customised version. Therefore, the origins for such results, based on empirical evidence, cannot be given.

Three participants (Participants 1, 2 and 5) needed less than 40 s (on average) to complete one task with the default version of the website. Two of them (Participants 1 and 2) were able to complete all eight tasks and found the correct answers by themselves when using the default version of the website. Both reported that the default version has an appropriate font size. Participant 5 needed help from the moderator when using the default website (75% success rate), because the website has overhead information on the one site. The other three participants (Participants 3, 4 and 6) needed less than 30 s (on average) to complete one task when using the customised version of the website. In addition, in this case, two participants (Participants 3 and 4) achieved a 100% success rate when using the customised website, while Participant 6 needed help from the moderator when using the customised website (88% success rate). However, all participants agreed on a better user experience and as the majority of them reported, this was due to appropriate contrast and font size.

#### 4.3.3. Satisfaction

**Figure 7:**
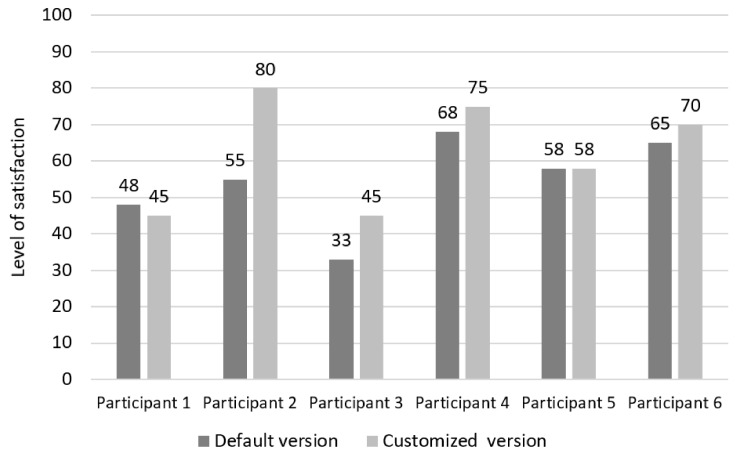
The results of satisfaction per participant.

Figure 7 presents the results of satisfaction per each participant. The satisfaction was measured and calculated by a System Usability Scale (SUS). The results are interpreted by Sauro’s reference limits [65]. On average, evaluations of both versions of the website were below average satisfaction, but a comparison between the versions show that participants evaluated satisfaction with customised website with 62 (± 15.17) scores, while the default version achieved 54 (± 12.724) scores. As illustrated in Figure 7, four participants (Participants 2, 3, 4 and 6) evaluated the customised version of the website with higher scores compared to the default version of the website, while one participant (Participant 1) assigned the scores in favour of the default website and one participant (Participant 5) expressed the same level of satisfaction with both versions. Additionally, three participants (Participants 2, 4 and 6) expressed above-average satisfaction with using the customised version of the website, while satisfaction for three participants (Participants 1, 3 and 5) was below-average. All participants evaluated the default version of the website with below-average satisfaction.

#### 4.3.4. Suitability

The average evaluations of each concept of suitability for both versions of the website are represented in Table 7. The answers were measured by the five-point Likert scale (where 1 indicates that the participants totally disagrees with the sentence, while 5 indicates that participants totally agrees with it). On average, participants agreed that the customised version of the website is in accordance with their needs and it is individually suitable due of colour contrast and content presentation, layout elements and navigation. Additionally, they agreed that it is easy to learn how to use the customised website (learnability), but they had neutral opinion about ease of use. The average evaluations of the default version of the website per each concept approximated a neutral opinion. Therefore, they reported that they neither agreed nor disagreed that the default website is suitable for them.

**Figure 8:**
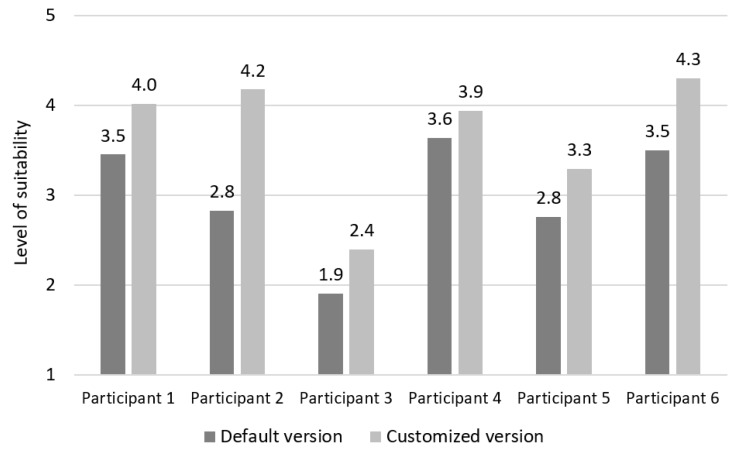
The evaluation of suitability per participant.

The participants’ opinions of suitability for both versions of the website are visible in Figure 8. On average, participants’ opinions of suitability for using the default version of the website was neutral (3 ± 0.7), while they agreed that the customised version of the website was suitable for them (3.7 ± 0.7). As can be seen in Figure 8, four participants (Participants 1, 2, 4 and 6) agreed that the customised version was suitable for them, while one participant (Participant 5) had a neutral opinion on the use of the customised version of the website and one participant (Participant 3) believed that the customised version of the website was not suitable for them.

Based on the interview questions, most participants (83%) expressed that they would like to use the individual website adjustments regularly in the future, while one participant (17%) reported using it occasionally.

### 4.4. Summary of Findings

**RQ1:** What parameters of individual website adjustments for people with dyslexia diverge or converge? 

In this research, none of the participants selected an equal combination of all three parameters of individual website adjustments. The most consistent selection was parameter “font type” (Arial, 67%), while the other two parameters (”font size” and “contrast between background and font”) were within the recommendations for people with dyslexia [20,29,38], even though the selections diverged between participants. Therefore, the results do support existing findings that there is no universal dyslexic web user [8] and that one universal design of a website is not suitable for people with dyslexia [15], because their needs and preferences are too diverse. 

**RQ2:** What are the benefits or challenges for people with dyslexia when interacting with adjusting a customised website? 

A detailed qualitative analysis was made, based on the participants’ comments during the tasks performed with both the default and customised website. As expected, the results show that participants experienced more challenges when using the default website, while they enjoying the most benefits when using the customised website. The issues identified with the default website are consistent with other existing research that has explored challenges with web interaction for people with dyslexia [37,40,41,42,43,44,45,46,47]. People with dyslexia pointed out the following issues when using the website that is not in accordance with their needs and preferences: unclear navigation, too much information on screen, not enough graphic elements, issues with visual appearance and inappropriately presented information. When using the customised website, issues of unclear navigation, not enough graphic elements and low visibility were reduced, while participants did not mention any issues with the visual appearance, too much information and inappropriately presented information. Therefore, this finding may indicate that the individual website adjustment can lead not only to reducing the issues, but also to eliminating some challenges that people with dyslexia have when they use the website. In addition, the customised website also provides benefits for participants, such as a better user experience for all participants and an appropriate contrast and font size for most of participants. All those benefits can have a positive impact on increasing web accessibility and web usability for people with dyslexia. 

**RQ3:** Do individual website adjustments for people with dyslexia yield any empirical advantages related to effectiveness, efficiency, satisfaction and suitability? 

The results of this research show that the participants archived an 81% success rate (effectiveness) with both the default and customised version of the website, while their average amount of task time was 47.31 s per task (efficiency) when using the default version of the website and 54.53 s per task (efficiency) when using the customised version of the website. The qualitative analysis of participants’ comments shows that the majority of the comments were made when the default version of the website was used (default version: 60% vs. customised version: 40%). Although the participants reported the majority of challenges when using the default website, they were still able to complete the four tasks by themselves. The participants that found the correct answer by themselves when using the customised version of the website reported that the website included the appropriate font size and contrast. Although the overall satisfaction level was evaluated as below-average (for both versions of the website), all of them agreed that they had a better user experience when using the customised website. Additionally, the suitability scores show that the customised website was appropriate for most participants, because it met their needs and preferences. When using the customised website, the navigation, i.e., moving around the web page and correcting mistakes, was easy for them, because it was clear how the screen elements worked. The appropriateness of the presented information (contrast, font type and size) contributed to better text readability, because the information was presented in a style that suited them. Therefore, the results indicated, that using the individual website adjustments for people with dyslexia are desirable in order to meet their needs and preferences.

### 4.5. Recommendations for Adapting Usability Evaluation Methods for People with Dyslexia

Based on the lessons learned during the usability evaluation, we recognised that the methods for usability evaluation, such as the thinking aloud protocol, log analyses, questionnaires and interviews do not need to be adapted for people with dyslexia, whereas we recognised that the adjustments to formal usability testing would be rational. The findings and recommendations for adapting formal usability testing for people with dyslexia are presented below.

The first recommendation is related to the technique for giving the tasks instructions. During the usability evaluation, it was found that people with dyslexia used their own specific reading approach. Overall, 67% of participants read the instructions on paper-based forms. The participants read instructions in separated segments and used their fingers to help them focus on the text. Based on these findings, it is recommended that the textual instructions be replaced with audio. Audio instructions would allow for multiple listens and allow the listeners to stop the record at a selected position, thereby allowing them to listen by segment. The option to facilitate the reading of the instruction given by the screen-based form for people with dyslexia is to allow them to use speech synthesis. This technique could improve the understandability of the instructions and reduce the cognitive effort of people with dyslexia prior to solving any tasks.

The second recommendation is related to the technique for acquiring task results. People with dyslexia can have a problem with reading aloud. Their reading strategy is often to first read the text quietly (sometimes even more than once), and read it out loud when then they get enough self-confidence to do so. Consequently, this reading strategy has an impact on measuring the time needed to perform a task. Furthermore, if people with dyslexia find the correct answer and read it incorrectly (because of their specific deficiencies), the task would be marked as unsuccessful. Therefore, the incorrect reading of the answer also has an impact on the metric of successful task completion. Based on these findings, the modified technique of giving the tasks’ answers is recommended. Therefore, people with dyslexia are offered the option of marking the task’s answers with a mouse. This provides a realistic time-frame to perform the task and avoid the incorrect reading of an answer, while people with dyslexia will not experience additional stress and personal distress.

The final important recommendation is related to the number of tasks. Based on the findings given by participants, we recommend that the number of tasks complies with the most essential functionalities that we want to investigate during the usability evaluation. The optimal number of task varies from five to eight.

### 4.6. Limitations and Future Work Directions

Some limitations need to be considered for this research. Primarily, the sample of experimental participants is difficult to generalise. The research included six participants of people with dyslexia. Although the sample size is within the recommendations for research with users with disabilities given by Lazar et al. [60], which emphasises that “it is generally acceptable to have 5–10 users with a specific impairment take part in a study” and according with Nielsen’s findings of sample size [57,58,59], further research will include a larger sample with people with different types and degrees of dyslexia. The next limitation is the parameters of website adjustment. This research focused on three main parameters, such as font style and size, background and font colour. The investigation of different/additional adjustment parameters (eg. character spacing, line spacing, paragraph spacing) may lead to different results. In addition, the website with integrated assistive technology included in this research has a specific design and structure. Therefore, the results and conclusions cannot be generalised to any website with integrated assistive technology, but can only be applied to websites with a similar design, structure and integrated assistive technology. The integrated assistive technology was composed by the limited and specific value collection of website adjustments for people with dyslexia. The extended or disparate value collection can lead to different results and to different conclusions.

Since people with dyslexia expressed a positive opinion about using individual website adjustments, the future work will be geared also into applied research. The focus will be directed on the development of extended assistive technology with additional sets of parameters and its advantages will be investigated on people with different types and degrees of dyslexia. Future work will be focused also on investigation of the usability evaluation with eye-tracking technologies. Based on the research performed by Rello and Ballesteros [67], we see the opportunity to develop the assistive technology based on automatically websites’ customisation for people with dyslexia. Besides, the future research will be focused on investigating the comparison of the usability of customised websites between people with and without dyslexia.

## 5. Conclusions

The purpose of web accessibility is to ensure that a website can be used by as many people as possible [1], regardless of their personal characteristics (e.g., age, knowledge, skills, disabilities, needs, demands and preferences). Although there are many general guidelines to ensure web accessibility, it is almost impossible to provide all needs, demands and preferences of heterogeneous user groups at the same time. Therefore, some experts recommend using a customisable web environment [9,16] with the possibility of website transformation [17,18] in accordance with users’ individual needs and preferences. This means that users have the opportunity to customisable the website by themselves in accordance with their individual needs and preferences.

A customised website could lead to an increase in web accessibility as well as in web usability for people with disabilities [18,22,23], including for people with dyslexia [9,24]. The existing literature shows that a universal design is not appropriate for everyone with dyslexia due to the individual profiles of people with different needs, demands and preferences [15]. Based on this, our study investigated how people with dyslexia react to a customised website that is in accordance with their individual needs and preferences in terms of their usability and suitability and compared their results with the usability and suitability of the default version of a website. The usage of website adjustments reduced and/or eliminated the challenges that people with dyslexia had when they used a website, which consequently might have a positive impact on increasing web accessibility for them. In addition, people with dyslexia expressed a positive attitude towards the use of future individual website adjustments.

Therefore, the website adjustments with a wide range of different sets of parameters may lead to improved accessibility and usability not only for people with dyslexia, but also for other end user groups irrespective of whether people have or do not have disabilities. This may be an opportunity for designers and developers to consider users’ needs and preferences and to strive to make websites with the possibility of transformations according to users’ needs and demands, either through customisation or personalisation.

## Figures and Tables

**Table 1:** Demographic participant’s characteristics.

Characteristics		f [%]
Gender	Male	50%
	Female	50%
Age	15–25 years old	50%
	26–35 years old	33%
	36–45 years old	17%
	46 or more years old	-
Frequency of computer use per week	0–9 h	-
	10–19 h	33%
	20–29 h	17%
	30–39 h	17%
	40 h or more	33%
Using a computer/laptop/tablet PC	1–5 years	17%
	6–10 years	33%
	11–15 years	33%
	16–20 years	-
	21 years or more	17%
Experience with using the Internet	1—An absolute beginner	-
	2	-
	3	67%
	4	-
	5—Very experienced user	33%
The most commonly used browser	Mozilla Firefox	-
	Internet Explorer	33%
	Chrome	67%
Knowledge of assistive technology	Yes	17%
	No	83%
Level of knowledge about assistive	Excellent	-
technology	Good	-
	Acceptable	
	Poor	67%
	Very Poor	-
	I do not know	33%
Use of assistive technology	Yes	33%
	No	67%
	I do not know	-
Frequency assistive of technology use	Daily	-
	Weekly	-
	Once a month	-
	A few times a year	33%
	Never	67%

**Table 2:** Individual website adjustments.

Website Adjustments	Stock Value	f [%]
Font size	14 pt	17%
	16 pt	17%
	18 pt	33%
	20 pt	-
	125% pt	17%
	150% pt	17%
	200% pt	-
Font type	Arial	67%
	Arial Bold	-
	Verdana	17%
	Verdana Bold	-
	Open dyslexic	17%
	Open dyslexic alt	-
	Century Gothic	-
Contrast (Font colour–background colour)	Black–White	17%
	Black–Beige	33%
	Turquoise–Black	17%
	Blue–Yellow	17%
	White–Black	17%
	Yellow–Blue	-
	Blue–White	-
	Black–Yellow	-
	Black–Violet	-
	Black–Green	-

**Table 3:** Participants’ benefits and challenges.

	Default Version		Customised Version	
Benefits		f [%]		f [%]
Appropriate font size		33%		67%
Appropriate contrast		-		83%
Better user experience		-		100%
Challenges		f [%]		f [%]
Use the search engine		67%		17%
Issue with visual appearance		50%		-
Too much information		67%		-
Not enough graphic elements		67%		33%
Content imperfection		33%		-
Content inconsistency		33%		-
Low visibility		50%		17%
Improper font type		33%		-
Improper contrast		33%		-

**Table 4:** Occurrence of participants’ comments during the tasks performed with the default and customised version.

Task	P 1	P 2	P 3	P 4	P 5	P 6	Freq.	%
Task 1	1	1	1	3	-	1	8	15%
Task 2	1	-	-	-	-	1	2	4%
Task 3	1	1	-	-	-	-	2	4%
Task 4	1	1	1	-	-	1	4	8%
Task 5	-	1	2	4	2	2	11	21%
Task 6	1	2	1	2	1	2	8	15%
Task 7	1	-	1	-	1	-	3	6%
Task 8	1	2	3	2	3	3	14	27%
SUM	7	8	9	12	7	9	52	100%

**Table 5:** Occurrence of participants’ comments during the tasks performed with the default version.

Benefits	Task 1	Task 2	Task 3	Task 4	Task 5	Task 6	Task 7	Task 8	Freq.	%
Appropriate font size	1	-	-	1	-	-	-	1	3	6%
Appropriate contrast	-	-	-	-	-	-	-	-	-	-
Better user experience	-	-	-	-	-	-	-	-	-	-
Challenges	Task 1	Task 2	Task 3	Task 4	Task 5	Task 6	Task	Task 8	Freq.	%
Use the search engine	1	-	-	1	2	-	-	-	4	8%
Issue with visual appearance	1	-	-	1	2	-	-	-	4	8%
Too much information	-	-	1	-	-	1	3	-	5	10%
Not enough graphic elements	-	1	-	-	2	1	-	-	4	8%
Content imperfection	-	-	-	-	1	1	-	-	2	4%
Content inconsistency	-	-	-	-	1	1	-	-	2	4%
Low visibility	2	-	-	-	-	1	-	-	3	6%
Improper font type	1	-	-	-	1	-	-	-	2	4%
Improper contrast	-	-	-	-	-	2	-	-	2	4%
SUM	6	1	1	3	9	7	3	1	31	60%

**Table 6:** Occurrence of participants’ comments during the tasks performed with the customised version.

Benefits	Task 1	Task 2	Task 3	Task 4	Task 5	Task 6	Task 7	Task 8	Freq.	%
Appropriate font size	1	-	-	-	1	-	-	4	6	12%
Appropriate contrast	1	-	-	-	1	-	-	3	5	10%
Better user experience	-	-	-	-	-	-	-	6	6	12%
Challenges	Task 1	Task 2	Task 3	Task 4	Task 5	Task 6	Task 7	Task 8	Freq.	%
Use the search engine	-	-	-	-	-	1	-	-	1	2%
Issue with visual appearance	-	-	-	-	-	-	-	-	-	-
Too much information	-	-	-	-	-	-	-	-	-	-
Not enough graphic elements	-	-	1	1	-	-	-	-	2	4%
Content imperfection	-	-	-	-	-	-	-	-	-	
Content inconsistency	-	-	-	-	-	-	-	-	-	-
Low visibility	-	1	-	-	-	-	-	-	1	2%
Improper font type	-	-	-	-	-	-	-	-	-	-
Improper contrast	-	-	-	-	-	-	-	-	-	-
SUM	2	1	1	1	2	1	0	13	21	40%

**Table 7:** The average evaluation of suitability per concepts.

	Default Version	Customised Version
Concepts	Mean (± SD)	Mean (± SD)
Attractiveness and colour suitability	2.8 (± 0.9)	3.7 (± 1.3)
Content presentation	2.7 (± 1.2)	3.9 (± 0.9)
Navigation and layout	3.3 (± 0.4)	3.7 (± 0.2)
Individual suitability	2.9 (± 0.7)	3.7 (± 0.7)
Ease of use	3.2 (± 0.4)	3.3 (± 0.6)
Learnability	3.2 (± 0.9)	3.9 (± 0.9)

## Appendix A

### Suitability Questionnaire

1. Attractiveness and colour suitability
  1. The website design is pleasant.
  2. The website design is stylish and attractive.
  3. The website has characteristics that make it especially appealing.
  4. The colours used throughout the website are appropriate.
2. Content presentation
  1. The website contains a good balance of graphics versus text.
  2. The pictures are understandable and unambiguously convey the information they represent.
  3. The icons are understandable and unambiguously convey the information they represent.
  4. The information is easy to read.
  5. The information is written in a style that suits me.
  6. Too little information is presented with graphic elements (e.g., picture, diagram).
  7. The typography (lettering, headings, and titles) is appropriate.
  8. Line spacing is appropriate.
3. Navigation and layout
  1. The navigation could be considered to be very easy.
  2. I moved between web pages without major problems.
  3. It is clear how screen elements (e.g., pop-ups, scrolling lists, menu options, etc.) work.
  4. The layout of elements on the website is clear.
  5. The content areas are clearly separated.
  6. The web page is attention-getting.
  7. Screens have the right amount of information.
4. Individual suitability
  1. The website was designed with me in mind.
  2. The website was designed in accordance with my needs.
  3. The website was designed in accordance with my demands.
5. Ease of use
  1. The website is easy to understand.
  2. Interacting with the website did not require a lot of mental effort.
  3. I would find it easy to get the information I needed from the website.
  4. The website was clear and understandable.
6. Learnability
  1. I can get to information quickly.
  2. The website is well-suited to first-time visitors.
  3. The website is well-suited to repeat visitors.
  4. I can easily remember how to use it.
  5. I could recover from mistakes quickly and easily.

## Appendix B

### Task Instructions

1. On which street is the organization located?
2. What is the working time of the organization?
3. What is the latest published news on the entry website?
4. Use the search engine on the website to find out how many hits the word “form” returns.
5. When was the Gambling Act published at the first time?
6. Find out when the latest news was posted to residents in connection with the annual assessment of personal income tax.
7. Find the form for entrance/exit from the European Union.
8. Subscribe to automatically receive news (RSS) from the “e-services”, by reading the ordering instructions first.
